# An Evaluation of Obstetric Characteristics and Contraceptive Use Among Refugee Women

**DOI:** 10.7759/cureus.24045

**Published:** 2022-04-11

**Authors:** Burcu Korkut, Nergiz Sevinç, Didem Adahan

**Affiliations:** 1 Department of Family Medicine, Karabük University Training and Research Hospital, Karabük, TUR; 2 Department of Public Health, Karabük University Faculty of Medicine, Karabük, TUR

**Keywords:** migration, obstetric history, contraceptive methods, women, refugee

## Abstract

Background

This study aimed to assess the obstetric characteristics of refugee women and evaluate their knowledge and usage of contraception methods.

Methodology

This retrospective, cross-sectional study included 400 married refugee women aged 18-49 years who presented to the Foreigners Outpatient Clinic between 2018 and 2020. In the Foreigners Outpatient Clinic, a health worker filled in a 23-question form for all refugee women to obtain their obstetric history and information regarding contraception methods. The 23-item form comprised 11 questions about the sociodemographic characteristics of refugee women, seven questions about their obstetric history, and five questions about their knowledge and attitudes about contraception methods. Statistical analyses were performed using the data obtained from these forms filed in the outpatient clinic. Descriptive data were presented as frequency, percentage distribution, mean, and standard deviation.

Results

The average age of the participants was 31.36 ± 8.36 years, with 52.8% of the participants being Afghan women. Overall, 70% of participants were either only literate, learned to read and write without ever going to school, or were primary school graduates. Moreover, 61.1% of refugee women aged ≤18 years at the time of first birth were Somali, Sudanese, and Saudi Arabian nationals, significantly outnumbering other refugee women (p = 0.03). The rate of having ≥three children among Pakistani participants was 90.0%, which was statistically significantly higher (p = 0.04). The proportion of Afghan women who received counseling on family planning was lower (p = 0.04). There was no statistically significant difference between refugee women’s knowledge of using a contraception method (p = 0.09). As a contraception method, the most significant use of injection was by refugee women from Somalia, Sudan, and Saudi Arabia (p = 0.03); tubal ligation was used by Afghan women (p = 0.01); and implanted by Pakistani women (p = 0.01). No difference was found in the use of condoms, pills, and intrauterine devices.

Conclusions

On evaluating the obstetric characteristics of refugee women, it was determined that the number of pregnancies and the rate of giving first birth at the age of 18 and under were high. On the other hand, there was no difference between refugee women regarding condom and pill use; however, it was observed that the rate of using these methods at some point in their lives was high. Hence, it can be concluded that immigration seriously affects women’s reproductive health, makes it difficult to obtain protection methods, and paves the way for having unplanned and large numbers of children.

## Introduction

Migration refers to the collective movement of people from their home country to settle in another place of residence due to reasons such as war, ethnic oppression, and economic crisis [1,2]. The main problems experienced by refugees include unfavorable living conditions, compulsory communal living, financial difficulties, language barriers, and inadequate access to effective and qualified healthcare services during and after their migration [3]. Globally, 55% of asylum seekers are people fleeing from such countries as Afghanistan, Somalia, Iraq, Syria, and Sudan due to civil war and conflict [4].

Due to its geopolitical location, Turkey encounters intense migration and has been exposed to a growing number of refugees, especially over the last decade, due to wars in the Middle East [5]. Approximately half of the refugees are women. In 2021, 3.6 million Syrians migrated to Turkey, of whom 1.6 million were women [6]. The factors that negatively impact refugee health affect female refugees the most due to their social status and traditional life patterns [7]. Furthermore, the combination of the culturally determined gender roles of women with patriarchal values leads to the prioritization of the needs of the entire family, pushing women’s sexual and reproductive health problems and needs to the background. The inevitable result is frequent and unhealthy pregnancies, recurrent miscarriages, and high maternal and infant mortality rates [8,9].

Given that refugee women should be educated in family planning and reproductive health, it is important to determine their reproductive health during their adaptation to a new life. Therefore, the purpose of this study was twofold: (1) to evaluate the obstetric characteristics of refugee women, and (2) to investigate their knowledge and usage of contraceptive methods. To our knowledge, to date, this is the first study evaluating obstetric characteristics and knowledge regarding contraceptive methods of refugee women from eight different countries.

## Materials and methods

Refugee women aged 18-49 years who applied to the Foreigners Outpatient Clinic (FOC) of the Karabük Community Health Center between January 2018 and January 2020 were included in this retrospective, cross-sectional study. Turkish women and refugee women with missing information on the migrant reproductive health form were excluded from the study. Written consent was obtained from refugee women for the use of data in scientific studies by paying attention to patient information confidentiality.

The study population comprised 400 refugee women all of whom were included in the study without sampling. A 23-question form was created by FOC healthcare professionals to monitor and protect the reproductive health of refugee women, in which obstetric history and prevention methods were queried. The form included 11 questions concerning sociodemographic characteristics questioning their age, educational status, nationality, and economic status. Questions about obstetrics stories included age at first birth, the total number of pregnancies, the number of live and stillbirths, and miscarriages. Finally, regarding the methods of protection, participants were questioned whether they received counseling services and whether they used methods such as pills, condoms, intrauterine devices (IUDs), implants, injections, and vasectomy at some point in their lives.

The data obtained from the archived information forms of refugee women who applied to FOC between 2018 and 2020 were transferred to SPSS version 25.0 (IBM Corp., Armonk, NY, USA) for statistical analysis. Mean, standard deviation (SD), frequency, and percentage values were used for descriptive statistics. Compliance of the data with normal distribution was determined by the Kolmogorov-Smirnow test. Data that did not fit the normal distribution were analyzed using the Fisher’s test as they were not suitable for the chi-square test. The statistical significance level was accepted as p < 0.05 for all analyses.

This study was approved by the Noninterventional Clinical Research Ethics Committee of Karabük University (Approval number: 2020/305; date: August 31, 2020). The Institutional approval was obtained from the Karabük Provincial Health Directorate.

## Results

The mean age of the refugee women was 31.36 ± 8.36 years, of whom 50.5% were aged ≥31 years. Of the refugee women, 52.2% were Afghan; 23.3% were Syrian; 12.0% were Iraqi; 5.5% were Iranian; 4.6% were from Somalia, Sudan, and Saudi Arabia; and 2.5% were Pakistani (Table 1).

**Table 1:** Distribution of refugee women aged 18–49 years by age, nationality, education, economic status, length of stay in Turkey, social security, and chronic diseases.

Demographic Characteristics	n	(%)
Age (years)		
18–24	17	4.3
25–30	181	45.2
≥31	202	50.5
Nationality		
Afghanistan	208	52.1
Syria	93	23.3
Iraq	48	12.0
Iran	22	5.5
Pakistan	10	2.5
Somalia, Sudan, Saudi Arabia	19	4.6
Education level		
literate	80	20.0
Primary school	200	50.0
Secondary school	60	15.0
High school	40	10.0
Higher education	20	5.0
Economic status		
Income less than expenses	208	52.0
Income equals expense	135	33.8
Income higher than expenses	57	14.3
Length of stay in turkey (years)		
<1	120	30.0
1–3	120	30.0
3–5	100	25.0
>5	60	15.0

There was a significant difference in the women’s age at the time of birth across nationalities (p = 0.03). The birth rate at the age of ≤18 years was the highest among the women from Somalia, Sudan, and Saudi Arabia (61.1%) and the lowest among women from Pakistan (20.0%). There was also a significant difference in the number of pregnancies between the nationalities of the women (p = 0.04). The rate of having ≥three pregnancies was higher among Pakistani women. The number of stillbirths varied according to the nationality of the respondents, with Syrian women having lower stillbirth rates (p = 0.01). No significant differences were found among nationalities in terms of the number of live birth, living children, and miscarriages (Table 2).

**Table 2:** Comparison of pregnancy and obstetric characteristics of refugee women by nationality. *: <0.05; Pearson’s chi-square test, Fisher’s test; **Other; Somalia, Sudan, Saudi Arabia

Patient characteristics		Nationality												P-value
		Afghanistan		Syria		Iraq		Iran		Pakistan		**Other		
		n	%	n	%	n	%	n	%	n	%	n	%	
Age at first birth (years)	≤18	98	46.9%	39	41.9%	19	39.6%	10	45.5%	2	20.0%	11	61.1%	0.03*
	19–29	105	50.2%	53	57.0%	27	56.3%	12	54.5%	8	80.0%	7	38.9%	
	30–39	6	2.9%	1	1.1%	2	4.2%	0	0.0%	0	0.0%	0	0.0%	
Number of pregnancies	1–2	61	29.2%	34	36.6%	11	22.9%	7	31.8%	1	10.0%	2	11.1%	0.04*
	≥3	148	70.8%	59	63.4%	37	77.1%	15	68.2%	9	90.0%	16	88.9%	
Number of live births	None	2	1.0%	0	0.0%	0	0.0%	0	0.0%	0	0.0%	0	0.0%	0.19
	1	29	13.9%	13	14.0%	5	10.4%	5	22.7%	1	10.0%	1	5.6%	
	2	72	34.4%	33	35.5%	21	43.8%	8	36.4%	4	40.0%	6	33.3%	
	≥3	106	50.7%	47	50.5%	22	45.8%	9	40.9%	5	50.0%	11	61.1%	
Number of living children	0	2	1.0%	0	0.0%	0	0.0%	0	0.0%	0	0.0%	0	0.0%	0.48
	1	30	14.4%	13	14.0%	6	12.5%	5	22.7%	1	10.0%	1	5.6%	
	2	75	35.9%	33	35.5%	21	43.8%	8	36.4%	4	40.0%	7	38.9%	
	≥3	102	48.8%	47	50.5%	21	43.8%	9	40.9%	5	50.0%	10	55.6%	
Number of miscarriages	None	149	71.3%	76	81.7%	28	58.3%	16	72.7%	5	50.0%	13	72.2%	0.12
	1	50	23.9%	12	12.9%	18	37.5%	5	22.7%	4	40.0%	5	27.8%	
	≥2	10	4.8%	5	5.4%	2	4.2%	1	4.5%	1	10.0%	0	0.0%	
Number of stillbirths	None	176	84.2%	89	95.7%	38	79.2%	16	72.7%	8	80.0%	15	83.3%	0.01*
	≥1	33	15.8%	4	4.3%	10	20.8%	6	27.3%	2	20.0%	3	16.7%	

Afghans received the lowest level of family planning counseling among refugee women (p = 0.04). The rate of using injections was significantly higher among refugee women from Other Nationalities (Somalia, Sudan, Saudi Arabia) (p = 0.03). The rate of using tubal ligation for contraception was significantly higher in Afghan refugee women (p = 0.01). The rate of implant use was statistically significantly higher in Pakistani refugee women (p = 0.01). The use of the withdrawal method, considered to be a traditional contraception method, was statistically higher among Syrian refugee women (p = 0.01) (Table 3). There were no significant differences among women of different nationalities regarding knowledge about contraceptive methods and usage of pills, condoms, IUDs, and vasectomy.

**Table 3:** Comparison of refugee women’s use of contraceptive methods by nationality. *: <0.05; Pearson’s Chi-square test, Fisher’s test; **Other: Somalia, Sudan, Saudi Arabia.

		Nationality												P-value
		Afghanistan		Syria		Iraq		Iran		Pakistan		**Other		
		n	%	n	%	n	%	n	%	n	%	n	%	
Counseling services	Yes	74	35.4%	37	39.8%	26	54.2%	8	36.4%	5	50.0%	9	50.0%	0.04*
	No	135	64.6%	56	60.2%	22	45.8%	14	63.6%	5	50.0%	9	50.0%	
Contraceptive methods information	Yes	82	39.2%	36	38.7%	20	41.7%	10	45.5%	4	40.0%	6	33.3%	0.09
	No	127	60.8%	57	61.3%	28	58.3%	12	54.5%	6	60.0%	12	66.7%	
Pills	Yes	183	87.6%	87	93.5%	45	93.8%	19	86.4%	10	100.0%	16	88.9%	0.06
	No	26	12.4%	6	6.5%	3	6.3%	3	13.6%	0	0.0%	2	11.1%	
Condoms	Yes	200	95.7%	86	92.5%	45	93.8%	21	95.5%	10	100.0%	18	100.0%	0.17
	No	9	4.3%	7	7.5%	3	6.3%	1	4.5%	0	0.0%	0	00%	
Intrauterine devies	Yes	118	56.5%	57	61.3%	27	56.3%	14	63.6%	5	50.0%	12	66.7%	0.15
	No	91	43.5%	36	38.7%	21	43.8%	8	36.4%	5	50.0%	6	33.3%	
Injections	Yes	98	46.9%	51	54.8%	24	50.0%	11	50.0%	4	40.0%	11	61.1%	0.03*
	No	111	53.1%	42	45.2%	24	50.0%	11	50.0%	6	60.0%	7	38.9%	
Tubal ligation	Yes	85	40.7%	21	22.6%	15	31.3%	5	22.7%	3	30.0%	6	33.3%	0.01*
	No	124	59.3%	72	77.4%	33	68.8%	17	77.3%	7	70.0%	12	66.7%	
Vasectomy	Yes	20	9.6%	9	9.7%	5	10.4%	5	22.7%	0	0.0%	1	5.6%	0.08
	No	189	90.4%	84	90.4%	43	89.6%	17	77.3%	10	100.0%	17	94.4%	
Implants	Yes	6	2.9%	2	2.2%	5	10.4%	1	4.5%	3	30.0%	3	16.7%	0.01*
	No	203	97.1%	91	97.8%	43	89.6%	21	95.5%	7	70.0%	15	83.3%	
Withdrawal	Yes	159	76.1%	73	78.5%	36	75.0%	16	72.7%	4	40.0%	12	66.7%	0.01*
	No	50	23.9%	20	21.5%	12	25.0%	6	27.3%	6	60.0%	6	33.3%	

## Discussion

This study revealed that Afghan refugee women had the highest rate of presentation to the FOC (52.1%). Overall, 70.0% of the women were literate or primary school graduates. On comparing the knowledge levels of refugee women regarding contraceptive methods, no statistically significant difference was found between nationalities.

Baş et al. (2018), in their study of Syrian refugees living in Edirne, found the mean age at first birth to be 19 years [10]. Gümüş et al. (2017), in their study of Syrian refugee women in Istanbul, reported that 68.4% of the participants delivered their first child before the age of 19 [3]. Baş et al. (2018), in their study of Syrian refugees living in Edirne, found the mean age at first birth to be 19 years [10]. Gümüş et al. (2017), in their study of Syrian refugee women in Istanbul, reported that 68.4% of the participants delivered their first child before the age of 19 [3]. Consistent with the above-mentioned studies, 61.1% of the participants in this study had their first birth before the age of 18. The reason for this is the dominance of the multi-child family tradition in refugees and the psychological effect of the migration phenomenon within considerations such as proliferation and existence.

The study by Akhavan and Lundgren found the quality of prenatal care was lower and low the rates of low birth-weight infants and stillbirths were higher among women who were taken refuge in Sweden from countries such as Somalia and Ethiopia than among Swedish women [11]. In this study, the rate of one or more stillbirths was found to be the highest among Iranian female refugees with a rate of 27.3%. This shows that refugee women experience similar reproductive health problems in different countries they migrated to. As a result, negative consequences such as miscarriage or stillbirth can occur.

A previous study by Dikmen et al. (2019) conducted in the Isparta province of Turkey reported that the number of living children ranged from one to four (55%) among refugee women [12]. The study by Gümüş et al. (2017) conducted in Istanbul found that 42.7% of the refugee women had ≥five children [3]. Similarly, in the present study, 40-50% of the female refugees presenting to the FOC had ≥three living children, with no difference between nationalities. There could be several reasons for this situation. The fact that contraception use is not common in Muslim countries is one of the reasons. There is a fear of being eliminated in the case of migration, and procreation can be perceived as the solution to this problem. On the other hand, in Middle Eastern countries, having a son is considered a sign of power. Therefore, the number of births can be high.

Dikmen et al. (2018) reported that Syrian refugee women living in Turkey have a low level of interest and education in contraception. They also stated that their husbands were as effective as the refugee women in the emergence of this situation [12]. Moreover, this study showed that Afghan women benefited the least from counseling services on contraception. Although the nationality rates of the studies conducted among refugee women vary according to the date and place of residence, the perspective on birth control methods remains the same. This may be due to the refugees’ similar ethnicity, low educational level, and the fact that the spouses of refugee women are not supportive of contraception.

The study shows that 58% of refugee women did not use any form of contraception. As the largest group in the study, Sudanese, Somali, and Saudi Arabian women had the least knowledge of contraceptive use. Gele et al., (2019) in their study on Somali refugees living in Oslo, found the rate of contraceptive use to be low due to religious beliefs [13]. The low birth control rates in this study may be related to the participants’ inability to benefit from counseling services adequately, lack of knowledge, and the perception that birth control is a violation of their religion.

Khasholian et al., (2017) in a study on Syrian refugees living in Lebanon, determined that women used the traditional calendar method [14]. In the study of Moroccan refugees in Spain, Alvarez-Nieoto et al. (2015) found that oral contraceptives were the most common method among refugee women, free of charge, in the country [15]. Salisbury et al. (2016) reported that almost none of the refugee women chose long-acting contraception [16]. Consistent with the above two studies, this study, methods of protection among refugee women, including injections, tubal ligation, implant use, and withdrawal, were different among the nationalities; however, no difference was found between the levels of condoms, IUDs, pills, and vasectomy.

Refugee women who immigrated to our country or other countries have a high rate of use of withdrawal or calendar method, a traditional contraception method. It may be because it does not require additional effort to reach the existing methods, the use of birth control methods is not considered important, and the level of education is low. In addition, the most commonly used contraceptive methods among modern methods are condoms and pills. This may be because access to existing methods is free.

This study has some limitations. This was a single-center, there was no translator in the FOC, and the education level of the spouses of refugee women was not specified among the sociodemographic characteristics. On the other hand, while many literature studies have been performed on Syrian refugees, the fact that Afghans constitute the largest part of our study is the strength of our research. Another limitation is the scarcity of studies on this subject, so the results were compared with a limited number of studies.

## Conclusions

In this study, in the obstetric histories of refugee women, a statistically significant difference was found between the nationalities of women aged 18 and younger regarding the number of births, the number of pregnancies, and the number of stillbirths. When the contraception status was evaluated, there were differences between the nationalities in the use of counseling services, tubal ligation, implant, and withdrawal methods of refugee women.

Research findings suggest that refugee women have difficulties in accessing health services. It is clear that refugee women, who are exposed to many victims due to economic, psychological, and social pressures, need more special precautions and services.
